# International consensus on sports, exercise, and physical activity participation during post-operative interventions for Adolescent Idiopathic Scoliosis: An e-Delphi study

**DOI:** 10.1371/journal.pone.0322346

**Published:** 2026-02-23

**Authors:** Susanna Tucker, Nicola R. Heneghan, Alison Rushton, Adrian Gardner, Emily Russell, Andrew Soundy

**Affiliations:** 1 School of Sport, Exercise and Rehabilitation Sciences, University of Birmingham, Birmingham, West Midlands, United Kingdom; 2 The Royal Orthopaedic Hospital NHS Foundation Trust, Birmingham, West Midlands, United Kingdom; 3 School of Physical Therapy, Western University, London, Ontario, Canada; 4 Aston Medical School, Aston University, Birmingham, West Midlands, United Kingdom; 5 Buckinghamshire Healthcare NHS Trust, Aylesbury, Buckinghamshire, United Kingdom; Iran University of Medical Sciences, IRAN, ISLAMIC REPUBLIC OF

## Abstract

**Introduction:**

Physiotherapists and surgeons have a significant role in promoting participation and offering a graded return to sports, exercise, and physical activity following spinal fusion in adolescent idiopathic scoliosis (AIS). However, there is a lack of evidence to guide post-operative rehabilitation and variability worldwide. This study aims to obtain consensus on 1) when it is safe and 2) how an individual with AIS might return to sports, exercise, and physical activity.

**Methods and analysis:**

An international electronic 3 round Delphi study was conducted and reported. Eligible expert surgeons or physiotherapists had either specific clinical or research experience in AIS. Round 1 included a series of open-ended questions, from which a series of statements were generated. Round 2 commenced with a summary of the existing literature for participants to review prior to rating statements on a 5-point Likert scale. Participants were also given the opportunity to make comments. Round 3 participants were asked to re-rate statements on the same 5-point Likert scale. Consensus was determined through content analysis of open comments >1 participant, for statements rated on the 5-point Likert >75% agreement (strongly agree or agree) were defined as having consensus, following round 3 Kendall’s coefficient of concordance was calculated to evaluate the strength of the agreement where >75% was achieved.

**Results:**

From 53 recruited participants (18 countries, 1 unknown), 41 responded to round 1, 32 to round 2, and 29 to Round 3 (14 surgeons, 15 physiotherapists). Round 1 generated 85 statements under 19 themes surrounding graded return to sports, rehabilitation milestones, philosophical approaches, and treatment modalities. Round 2 generated 56 statements, > 75% with seven split into two due to multiple concepts, yielding 63 statements across 9 themes with >75% agreement. Themes included overarching considerations of care, the MDT, physiotherapy treatment modalities, pre-operative care, inpatient stay, and post-operative rehabilitation phases 1, 2, 3, and 4. Round 2 open comments generated a further 22 statements. Round 3 generated 66 statements with >75% agreement across the same 9 themes. All round 3 statements demonstrated significance (p < 0.001) with moderate agreement (W = 0.5). A Wilcoxon Sum-rank result (p < 0.05) showed stability between rounds 2 and 3. An additional 5 recommendations were generated from round 3 open comments exploring types of post-operative exercise, provision of rehabilitation, timeframes and milestones, and MDT involvement.

**Conclusion:**

This Delphi study provides the first international consensus of 71 statements on return to sports, exercise, and physical activity following spinal fusion in AIS. However, further subgroup analysis demonstrated consensus among surgeons and divergence among physiotherapists highlighting the need for further exploration of these statements.

## Introduction

Adolescent idiopathic scoliosis (AIS) affects approximately 2–3% of the population and is characterised by a complex three-dimensional spinal deformity [1,2]. Typically, around 10% of individuals with AIS, and with curves that measure above 50^0^, will go onto have spinal fusion. Biopsychosocial rehabilitation forms an essential part of recovery [3–7].

Following spinal fusion in AIS many individuals experience a reduction in muscle power, reduced range of motion and flexibility, decreased musculoskeletal function, and overall deconditioning [8,9]. Additionally, individuals have reported an increase in pain and fear causing between 28.0 to 36.6% of individuals to choose lower impact activities post-operatively [9]. Following surgery 32.2% do not return to pre-operative activities [10]. However, movement and activity post-operatively have been shown to reduce ongoing disability, dissatisfaction, societal and economic costs as well as the requirement for further operative intervention [11]. Furthermore, psychological interventions delivered post-operatively improve pain, anxiety, quality of life, and satisfaction [12,13].

Both physiotherapists and surgeons have a role in promoting post-operative return to sports, exercise, and physical activities [11,14]. Furthermore, physiotherapeutic interventions have been demonstrated as essential in promoting return to function in lumbar fusion [11]. Pre-operative ambulation exercises in AIS have been shown to improve post-operative recovery [15]. Immediate post-operative rehabilitation has been well documented in AIS such as strengthening, flexibility, and early day 1 mobilisation improving pain, recovery, and an earlier hospital discharge [16–19]. A recent Delphi study explored surgeon consensus on post-operative care following posterior spinal fusion in AIS, but focused on interventions used in the acute phase during hospital stay rather than during outpatient rehabilitation [20]. There is very little evidence for rehabilitation after an individual has been discharged from the acute setting [9,11].

The International Society On Scoliosis Orthopaedic and Rehabilitation Treatment (SOSORT) supports the inclusion of the wider multidisciplinary team (MDT) in post-operative care [12]. However, there remains a lack of consensus regarding which MDT members are necessary in the return to sports, exercise, and physical activity post-operatively [21,22]. At present most physiotherapeutic outpatient care remains surgically guided with variability worldwide seen between surgeons and between departments [21,23]. There is documented variability between physiotherapists regarding the reported rehabilitation interventions that are deemed to be most effective, alongside an array of biological, psychological, and sociological adjuncts to care [12,24–28]. Furthermore, there is evidence to suggest variability and a lack of consensus in care on a variety of issues such as rehabilitation milestones, post-operative protocols, and at what point it is necessary or safe to commence exercise and return to sports, exercise, or physical activity [9,11,23]. This lack of consensus has resulted in a large spectrum of different rehabilitation approaches amongst those involved in the management of AIS, ranging from highly conservative immobilisation to early mobilisation and return to sports, exercise, and physical activity [23]. This lack of clarity in rehabilitation has resulted in mixed expectations amongst medical professionals, patients, and caregivers, patients feeling fearful to move, participate in physical education classes at school, sports clubs, and ultimately lacking understanding regarding their care [10,23].

Consequently, a study establishing consensus in post-operative rehabilitation and return to sports, exercise and physical activity is required. Return to sport can be influenced by a large number of factors such as parental influence, socioeconomic, and psychosocial variables [10]. Furthermore, rehabilitation interventions might contain a spectrum of different biopsychosocial factors including restoring normal movement patterns and the promotion of self-management, education and advice, exercise, physical activity, or sports participation [7,29,30]. Therefore, research that addresses this area must consider specific concepts such as the content of any rehabilitation, the philosophical approaches used and the milestones achieved for the longer-term outpatient physiotherapeutic interventions that promote a return to sports, exercise, and physical activity. Consideration of the role of the MDT will also be key in understanding post-operative rehabilitation.

### Aim

To determine global surgeon and physiotherapy expert consensus on the rehabilitation milestones and philosophical approaches taken in the return to sports, exercise and participation in physical activity for the postoperative care of those with AIS who have undergone spinal fusion surgery.

### Objectives

1. To determine surgeon and physiotherapy expert consensus on timelines and milestones in returning to sports, exercise and physical activity participation in AIS.
2. To determine expert consensus on the involvement of the MDT and philosophical approaches used for the postoperative rehabilitation interventions for AIS.

## Methods

### Study design

This international three round electronic Delphi study was completed between February and November 2024. This study was performed according to a published open access protocol and is reported in accordance with the Conducting and REporting Delphi Studies (CREDES) guidelines [31–33].

### Sample and expert eligibility

This Delphi survey was distributed to a snowball sample of surgeon and physiotherapy experts working internationally within the management of AIS.

A sample of surgeon and physiotherapy experts from two populations were recruited [34]:

1. Physiotherapy or surgeon academics with ≥2 publications on AIS, paediatric spinal pain, or adolescent spinal pain in peer reviewed journals within the last 10 years.
2. Clinical surgeon or physiotherapy experts with a caseload of ≥20% spinal deformity practice specifically in AIS (either conservative or operative) per month.

### Recruitment

Recruitment took place from 9^th^ February 2024–11^th^ June 2024. Participants were contacted via scoliosis professional network calls, publicly available non-NHS emails, and social media snowballing [35]. The survey was sent electronically to individuals from a range of diverse backgrounds and a variety of geographical locations [31,36]. Professional networks included the British Scoliosis Society, Scoliosis Support and Research, Scoliosis Australia, Straight Caribbean Spine Foundation, Spine & Scoliosis Research Associates Australia, Scoliosis Research Society, National Scoliosis Foundation, Bundesverband Skoliose- Selbsthilfe, Vereniging van Scoliosepatiënten, Verein Scoliose Schweiz, Setting Scoliosis Straight, International Society On Scoliosis Orthopaedic and Rehabilitation Treatment, and AO Spine.

### Sample size

In order to achieve consensus, a minimum of 27 experts were required to complete all three rounds [37–41]. This was based on the protocol sample size considerations [42]. This calculation adheres to the CREDES guidelines regarding heterogeneity of expert groups [31]. Due to an estimated response rate of 70% this study aimed to recruit a minimum of 40 consenting experts (20 surgeon and 20 physiotherapy experts) to ensure the minimum number of respondents [37–40,43,44]. To help reduce risk of drop-out, experts chosen were willing individuals who have interest and subject specific knowledge rather than enticement through monetary reward [36,45–48].

### Procedure

**Fig 1:**
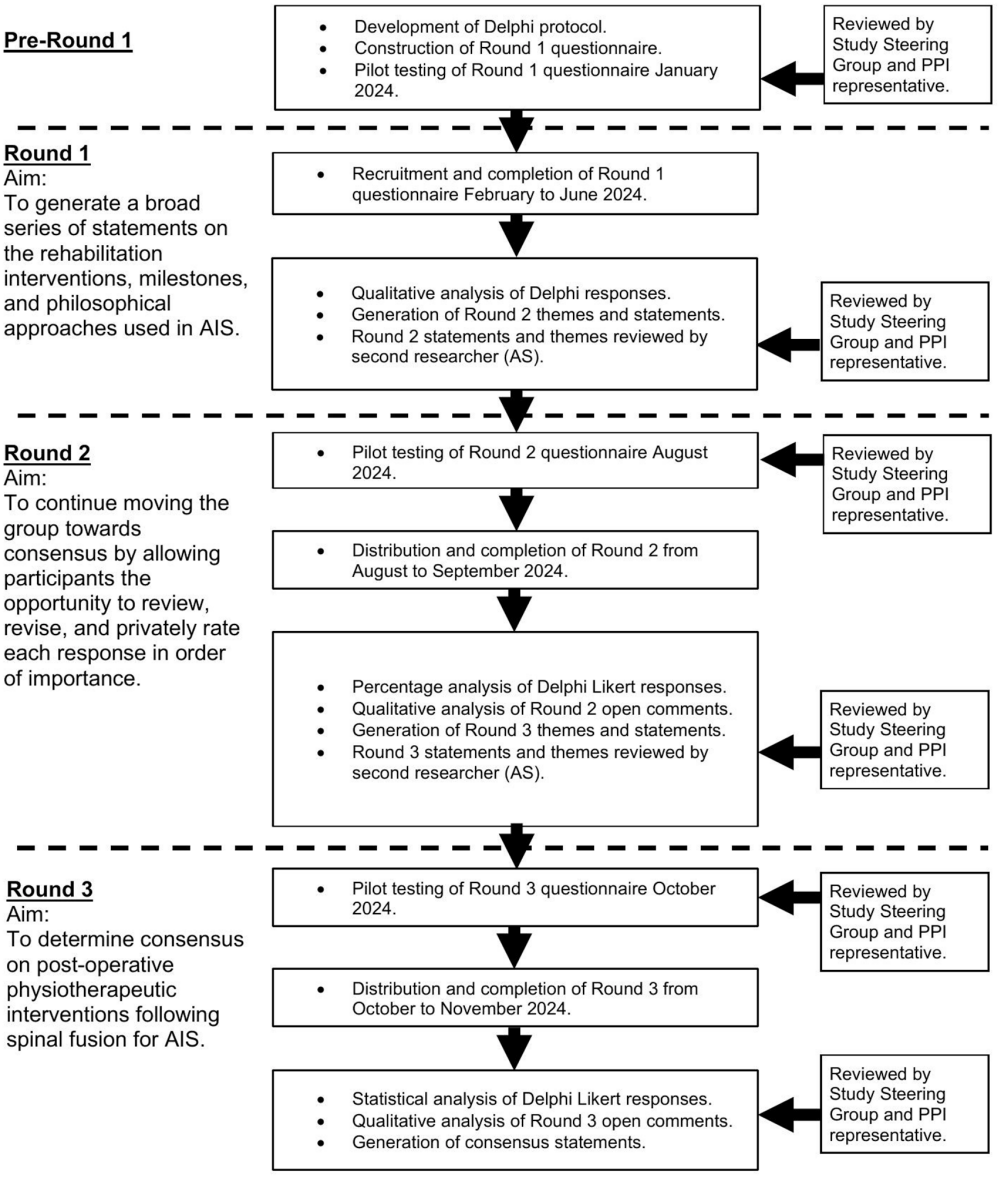
Stages of Delphi procedure.

This Delphi study consisted of three iterative rounds. Fig 1 shows a summary of the Delphi rounds.

### Pilot testing

Round 1 was piloted with 1 physiotherapist and 1 surgeon from a tertiary hospital specialising in the care of those with AIS, both participants who completed the pilot testing of rounds were not included in the main study [36]. Piloting of the round 1 survey took place in January 2024 prior to distribution of the round 1 survey. All 3 rounds were piloted with the same two individuals [36].

### Round 1

The aim of round 1 was to generate a broad series of statements. The study steering group (AS, NH, AG, AR) and PPI representative (ER) reviewed the questionnaire pre- and post-piloting of the survey [49,50]. During round 1 individuals were asked a series of open-ended questions exploring rehabilitation interventions, milestones, and philosophical approaches used in AIS, and asked to anonymously express their views with freedom in responses (data in S1 File) [51,52]. Round 1 did not include any supplementary material or prior reading to reduce bias [33,53]. Data were also gathered on the individual’s geographical location, percentage of practice involving spinal deformity, and nature of either clinical or academic work.

Inductive content analysis was used to identify themes, patterns, or concepts [53,54]. All results were verified by a second researcher (AS) and reviewed by the study steering group to ensure that data was represented fairly [52]. Dissonance was highlighted for consideration in round 2 in an attempt to move towards whole group consensus [55]. Data on participant characteristics including location and volume or nature of work was also tabulated to aid the analysis of results and illustrate any potential confounders or mediators in results analysis. Consensus during round 1 was defined as agreement >1 participant for responses given.

### Round 2

The aim of round 2 was to continue moving the group towards consensus by allowing participants the opportunity to review, revise, and privately rate each response in order of importance [35]. All participants from round 1 were invited to take part in round 2, including those who did not complete round one, to help reduce the risk of false consensus [56]. A systematic review of the current literature on factors that influence participation in sports, exercise and physical activity in paediatric spinal pain was introduced prior to Round 2 data collection to promote efficiency and guide participants towards the topic of interest [7,31,36]. This seeded approach is supported, efficient, and helps standardise the knowledge base [31,36,57]. In order to reduce bias from pre-existing literature only ‘open access’ articles were included [36]. Ideas and statements generated during round one with agreement >1 were re-distributed during round 2 with any dissonance between the physiotherapy and surgeon experts summarised. During round 2 participants were invited to rate their agreement with statements using a 5-point Likert scale, 1 = strongly disagree, 5 = strongly agree. Participants were also given the opportunity to offer their opinion in an open text box in response to themes and statements redistributed.

Likert rated numerical data from round 2 was quantitatively analysed using descriptive and inferential statistics [52]. Statements rated with either agree or strongly agree were used to create a percentage to evaluate consensus amongst experts for each statement [49]. Although there is some literature to suggest dichotomising ordinal data may reduce the accuracy of other data sets and instead recommend models such as a Markov Random Field Model this does not provide a percentage distribution, therefore was decided against for this Delphi [58]. Other Delphi studies have successfully reported dichotomising or trichotomizing a 5-point Likert scale into agree and disagree (and neutral) when calculating percentage agreement with agreement necessary for consensus documented between 60% and 80%, with 75% agreement recommended [55,59–64]. Central tendencies of rated items (median) and dispersion (interquartile range) were calculated summarised at the start of round 3 [52,55]. Any open-ended responses that had agreement across >1 participant were grouped and summarised for inclusion in round 3 once verified by the second researcher (AS) [31,52,55]. Consensus during round 2 was determined when >75% participants within the group agreed with a statement [65].

### Round 3

The aim of round 3 was to determine consensus on post-operative physiotherapeutic interventions following spinal fusion for AIS. Participants were provided with a summary of the results from round 2 with areas of dissonance identified, no further pre-existing literature was included. Summary data including descriptive and inferential statistics, dissonance, and stability [50,52] were visible to all consenting participants. Those who have withdrawn, regardless of their central tendency or dispersion, were excluded [51]. Participants were asked to rate their agreement with the statements that achieved >75% agreement during round 2, using the same 5-point Likert scale but with an additional opportunity for open comments.

Round 3 rated numerical data was quantitatively analysed in the fashion already described for round 2 with statements >75% agreement put forwards for further statistical analysis. Round 3 statements were statistically analysed using Kendall’s coefficient of concordance [66]. Kendall’s coefficient is designed to statistically analyse strength of agreement, rather than to determine exact cut off points [67–70]. Stability between round 2 and 3 was calculated using a Wilcoxon rank-sum test with stability inferred by significance at P < 0.05, making unlikely that stability was achieved due to chance [50,55,71–74]. The Wilcoxon rank sum was chosen over the signed rank test due to our data being from 2 independent rather than matched samples, all consenting and eligible participants were invited to Round 2 and 3 regardless of whether they had completed previous rounds to reduce false consensus [55,56]. Consistency and stability between responses between Rounds was considered a necessary criterion for stopping [55]. Data was tabulated and presented summarising results and consensus on post-operative interventions for return to sports, exercise, and physical activity. During round 3 there was a final opportunity for open comments. Comments made >1 participant were formed into recommendations. However, these final statements were not rated by all participants on the Likert scale and therefore have not been subject to statistical testing from Kendall’s W or Wilcoxon sum rank test for stability. These additional recommendations have been recorded in a separate table to provide clarity for the reader.

### Definition of consensus, agreement and stability

During each round agreement was assessed to determine the presence of consensus and progression into subsequent rounds (Table 1). Agreement was defined as (a) the level of occurrence >1 for qualitative statements or (b) >75% occurrence for items rated strongly agree or agree on the 5 point Likert scale between expert participants, between expert participants, thereby enabling prediction of the rating of another expert [55]. Stability between rounds 2 and 3 was tested and defined as consistency of responses amongst the group for each statement using a Wilcoxon sum-rank test [49,55,72]. Final consensus refers to Round 3 statements with both >75% participants rating items as strongly agree or agree, stability as determined by Wilcoxon sum rank test, and the strength of this consensus has been statistically analysed using Kendall’s coefficient of concordance [49,55,67,72]. The p-value for Kendall’s coefficient of concordance is a probability that measures the evidence against the null hypothesis, a p < 0.05 indicates significance with stronger evidence against the null hypothesis, making it unlikely that agreement was achieved due to chance [73,75].

**Table 1:** Criterion for progression.

Item	Criterion for Progression
**Consensus for open ended qualitative comments**	Agreement >1 participant for qualitative comments made.
**Consensus for Likert rated statements Round 2 and 3**	Agreement ≥75%, either strongly agree or agree,between participants for each statement and stability using a Wilcoxon sum-rank test [49,55,72].
**Strength of Consensus on final Round 3 statements**	Strength of consensus was statistically analysed using Kendall’s coefficient of concordance; Unusually strong agreement = 0.9, Strong Agreement = 0.7, Moderate Agreement = 0.5, Weak Agreement = 0.3, Very Weak Agreement = 0.1 [67–70]. [67–70]

### Data management

All rounds of both the pilot and the final Delphi were completed using Research Electronic Data Capture (REDCap) software for electronically distributing and collecting survey data [76,77].

The three rounds of the Delphi study distributed to surgeon and physiotherapy experts is summarised in Fig 1. Participants did not meet directly during this study but instead were sent online questionnaires seeking views on the topic of interest [51]. The survey and survey data was distributed, collected, stored and analysed electronically using REDCap [77]. REDCap is a secure online software that is designed for creating and managing online surveys [78]. All data is securely stored using REDCap on a password-protected computer with members of the research team only having access to the data. Following completion of the study, data will be securely stored within the University of Birmingham, UK for 10 years following which it will be safely destroyed in accordance with the University of Birmingham guidelines.

### Study steering group

Co-authors (AS, NH, AG, AR) constitute the study steering group, comprising methodological, academic expertise and clinical expertise in physiotherapy and spinal surgery. The group met to discuss the results analysis from each round and iteration in the subsequent round and to provide feedback and critical insights on the progress of the project.

### Patient and public involvement (PPI)

A PPI representative (ER) has been involved from study conceptualisation until final dissemination. Both clinicians and academics working within the field of AIS were involved in making research decisions and giving feedback regarding methodology and results synthesis at all stages of the process. The Guidance for Reporting Involvement of Patients and the Public short form checklist (GRIPP2-SF) has been used to promote PPI reporting [79] (data in S1 File).

### Ethics and dissemination

Full ethical approval has been provided by the University of Birmingham, Reference number: ERN_1617-Nov2023. Dissemination will take place through conference presentation and peer reviewed publications.

## Results

Fifty-three eligible experts consented to participate in this study (27 surgeons and 26 physiotherapists) (Table 2).

**Table 2:** Demographic Data.

Characteristics	Number of Participants	Characteristics	Number of Participants
**Profession**		**Years of Experience**	
Surgeon	26	1-5	3
Physiotherapist	27	6-10	6
**Geographical Location of Practice**		11-15	15
Australia	5	16-20	9
Brazil	1	21-25	7
Canada	3	26-30	6
Egypt	1	31-35	0
France & Italy	1	36-40	2
Greece	1	41-45	2
Ireland	1	46-50	1
Italy	2	51-55	1
India	1	Unknown	1
New Zealand	2		
Netherlands	3		
Malaysia	1		
Saudi Arabia	1		
South Korea	1		
Spain	2		
Turkey	2		
United Kingdom	16		
USA	8		
Unknown	1		
**Percentage of Working hours in Spinal** **Deformity Practice**			
0-20%	2		
20-40%	7		
40-60%	11		
60-80%	13		
80-100%	20		
**>2 publications in AIS within the last 10 years**			
Yes	31		
No	21		
Unknown	1		

### Participant demographics

Demographic data regarding profession, geographical location, experience, and practice is summarised (Table 2). There were 26 surgeons (49%) and 27 physiotherapists (51%). Experts were from 18 different countries (1 unknown) spread across all 5 continents. Fifty-one (96%) participants worked >20% of their hours in spinal deformity practice, 2 participants (4%) worked less than 20% of their hours in spinal deformity practice. However, these 2 participants were both eligible to participate in the e-Delphi for having >2 publications within last 10 years.

### Round 1

**Fig 2:**
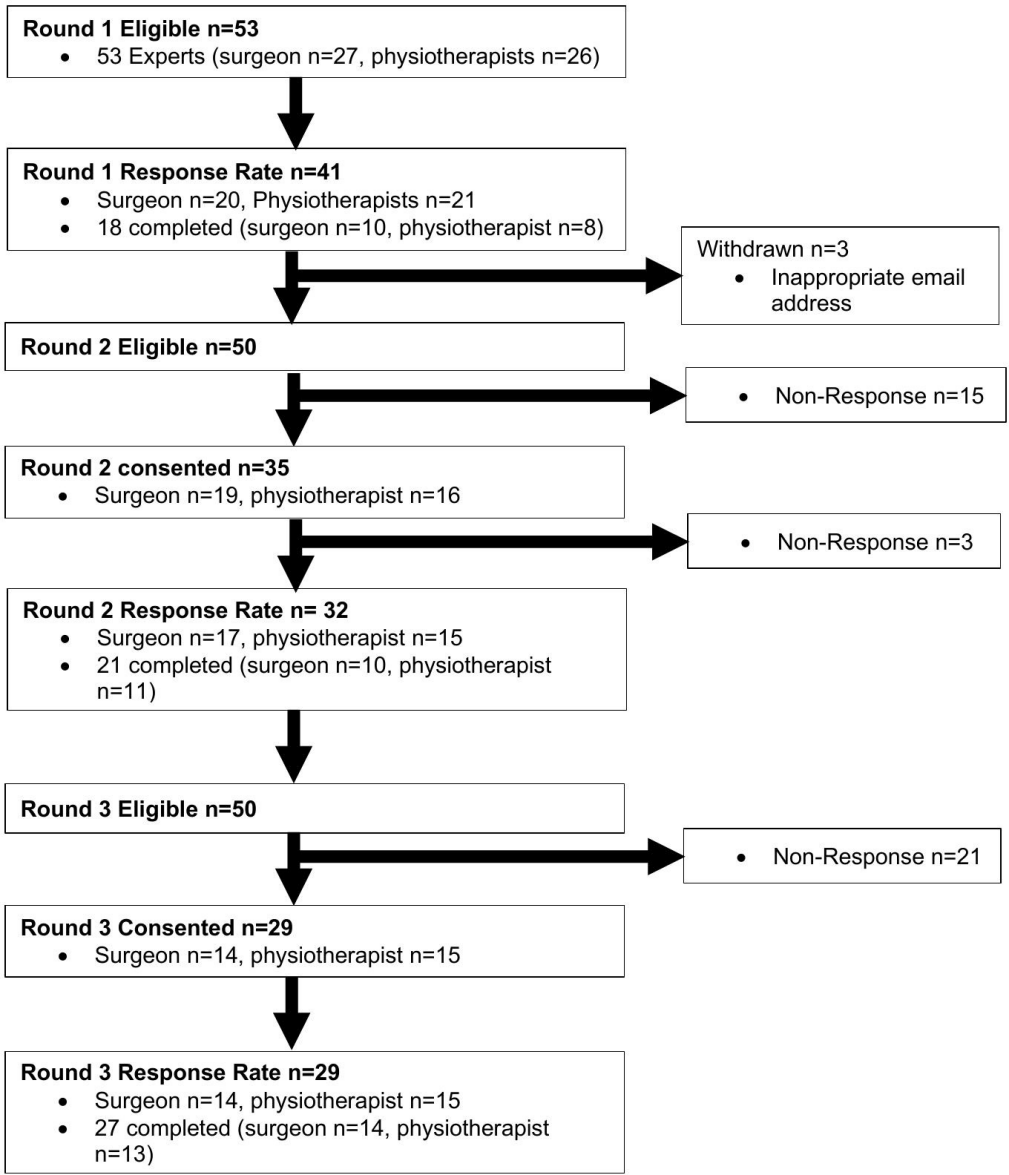
Flow Chart illustrating Delphi participants.

Fifty-three participants were included in the study because they were both eligible and gave consent (from the initial 60 participants who gave consent). Forty-one out of fifty-three experts (77%) commenced Round 1 and eighteen experts completed all questions in Round 1 (Fig 2). During Round 1, 85 statements were generated with agreement >1 participant. These 85 statements were grouped under 19 themes and redistributed during round 2 for Likert rating data in S1 Appendix.

### Round 2

**Fig 3:**
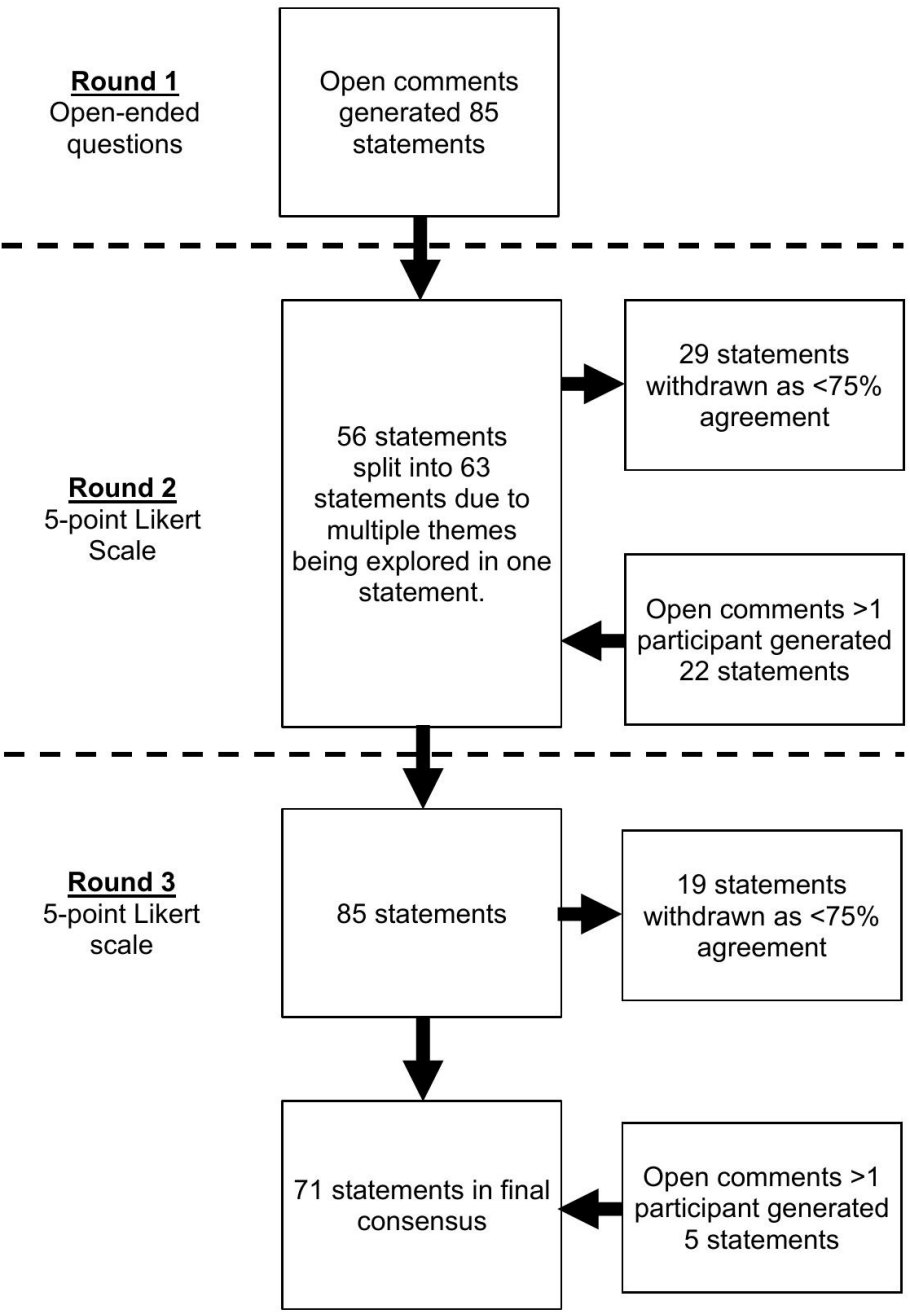
Statements generated during Delphi process.

Thirty-five of the fifty eligible experts consented to complete Round 2 and 32 participants commenced Round 2. Of these twenty-one completed Round 2. During Round 2, 56 out of 85 statements reached > 75% agreement on the 5-point Likert scale (5 = Strongly Agree or 4 = Agree) (data in S2 Appendix). Of the 56 statements agreed in round 2 seven explored multiple concepts and each of these seven statements were split into two statements making a total of 63 statements put forwards into round 3 (Fig 3).

Despite multiple data checks there were four statements progressed to Round 3 with <75% agreement due to a processing error. However, this was visible to all participants during round 3 with bar charts showing levels of agreement. During Round 3 two of these four statements (Strengthening Exercises and Cardiovascular Fitness modalities) were then included in the final consensus due to achieving >75% agreement in Round 3. Meanwhile, two statements regarding a cognitive behavioural approach and commencing water-based exercise/ rehabilitation or hydrotherapy as soon as the wounds are healed (approx. 2 weeks post-op) both scored <75% agreement therefore were removed from the final consensus. These 4 statements are highlighted in S2 Appendix. Quality control checks to prevent similar errors include data checking by a second researcher (AS) and sharing of data with study steering group throughout project.

Open comments from Round 2 were analysed and where there was consensus (frequency >1 participant) on a comment or theme an additional statement was generated (data in S2 Appendix). Interestingly, Round 2 open comments generated 2 statements regarding hydrotherapy or water-based exercise highlighting dissonance on opinion of the role of water or hydrotherapy (data in S2 Appendix). In total there were 22 additional statements generated from Round 2 open comments. In total 85 statements were put forwards for rating in round 3. These statements were arranged into 10 themes regarding different aspects of care.

### Round 3

During Round 3, 29 participants partially completed the survey (14 surgeons and 15 physiotherapists) and 27 participants answered all questions and completed to the end of Round 3 (14 surgeon, 13 physiotherapists) (Fig 2). A total of 85 statements were put forwards into round 3 as 7 of the 56 original statements were split into 63 due to multiple concepts being explored, and 22 statements generated from Round 2 open comments (data in S1 Appendix). Of the 85 Round 3 statements, 66 achieved >75% agreement (5 = Strongly Agree or 4 = Agree). For the 66 statements rated using the Likert scale, Kendall’s Coefficient of Concordance (W) was calculated at 0.5 moderate agreement on all items rated 29 and 27 participants respectively (Table 3) giving fair confidence in ranks [67]. As part of exploring dissonance the differences in consensus between surgeons and physiotherapists were evaluated (Table 3). Both groups demonstrated significance in the results, but the surgeons had greater agreement (W = 0.5, p < 0.001) compared with physiotherapists (W = 0.2, P < 0.001). The Wilcoxon sum rank test demonstrated stability with P > 0.05 across all 63 statements.

**Table 3:** Inter-rater Kendall’s coefficient of concordance for round 3 statements.

Round 3 Statements	All Participants		Surgeons		Physiotherapists	
	W	P	W	P	W	P
All 85 Round 3 statements rated Strongly Agree or Agree	0.442	<0.001	0.456	<0.001	0.465	<0.001
Final 66 Round 3 statements that had > 75% Strongly agree or agree	0.469	<0.001	0.473	<0.001	0.191	<0.001

Percentage agreement for areas of dissonance among physiotherapist has been detailed below in Table 4.

**Table 4:** Areas of dissonance among physiotherapists.

Statement	Percentage Distribution			Number of physiotherapist participants rating
	Strongly Agree or Agree	Neither Agree nor Disagree	Disagree or Strongly Disagree	
Patients ought to avoid maximal/ extreme flexion or bending movements post-op. Most common reasoning identified a need to establish that the fusion was stable and to prevent unnecessary pain.	73.3	26.7	0.0	15
Frameworks and their principles do not need to be formalised/ labelled	46.7	26.7	26.7	15
A pre-admission home exercise plan ought to be offered to optimize function and aid return to sports, exercise, and physical activity with muscular benefits identified.	71.4	14.3	14.3	14
Scoliosis Specific Exercises ought to be used in post-operative rehab	50.0	7.1	42.9	14
Usual care consists of approximately 5 days in hospital with the discharge criteria including pain control and bowels opened.	46.2	30.8	23.1	13
Do not routinely offer respiratory exercises or incentive spirometry.	30.8	15.4	53.8	13
Intermediate phase rehabilitation from 4–6 months ought to be part of post-operative care.	61.5	23.1	15.4	13
A late-stage rehabilitation component ought to be offered from 12 weeks onwards	61.5	23.1	15.4	13
A late-stage rehabilitation component ought to be offered from 12 weeks onwards	61.5	30.8	7.7	13
A 3–4 month review post-op needs to be offered addressing stiffness, shoulder pain, and function – physiotherapy may be required	53.8	30.8	15.4	13
Post-op reviews can be completed by other members of the MDT such as ACPs or Nurses	38.5	30.8	30.8	13
A cognitive behavioural approach ought to be taken in post-op care in AIS. This may aid behaviour change and postural correction.	61.5	30.8	7.7	13
A supported MDT view of return to sports after 6 months	53.8	38.5	7.7	13
Post-op Surgeon review at 12 months is important. This may help with identifying any further concerns, or possibly lead to discharge from surgical care.	69.2	15.4	15.4	13

**Fig 4:**
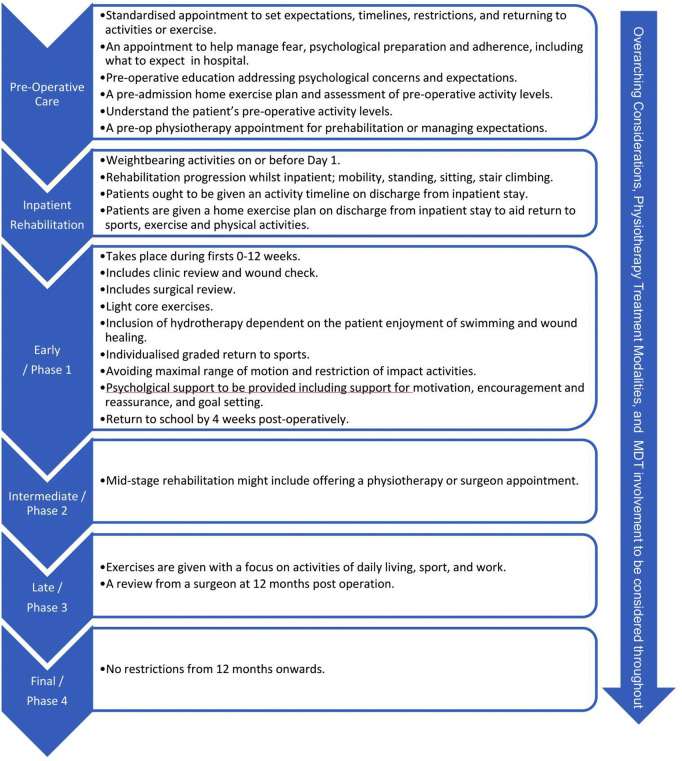
Narrow focus summary of Delphi consensus.

During Round 3, 66 consensus statements were agreed upon (Table 5) with five additional Round 3 recommendations from open comments (Table 6) making a total of 71 statements. Within the final 71 statements 14 out of the 22 round 2 additional comments resulted achieved consensus (Fig 3). The 66 statements that achieved consensus during round 3 comprised of 9 themes: (1) Overarching considerations, (2) Pre-operative Care, (3) Physiotherapy Treatment Modalities, (4) MDT involvement, (5) Inpatient Rehabilitation, (6) Early/ Phase 1 Care, (7) Intermediate/ Phase 2 Care, (8) Late/ Phase 3, and (9) Final/ Phase 4. The theme of overarching considerations and physiotherapy treatment modalities are not restricted to a certain time point/ stage of the process from decision to undergo scoliosis correction via spinal fusion, but rather are components of care that may be used at any stage in the process. All statements regarding components of care that achieved consensus are listed in Table 5 related to their respective themes. Fig 4 demonstrates the statements in a form that may provide appropriate guidance for healthcare professionals.

**Table 5:** Consensus Themes, Concepts, and Statements.

Theme	Concept	Statements
Overarching Considerations	This theme included statements on different aspects of care that are not necessarily time or milestone based. These overarching considerations ought to be considered throughout the rehabilitation process. Considerations that achieved consensus included frameworks such as patient centered or biopsychosocial care, using milestones in rehabilitation, graded lifting, carrying and return to sports, returning to school, promoting exercise, and awareness of an individual’s psychological state.	A graded return to sports (RTS) is important and beneficial in progressing up to contact sports, promoting recovery and function. This could be milestone based or time based.
		Time based milestones are an important consideration in physiotherapy progression. Milestones described ranged from 4 weeks to 12 months.
		Patients ought to avoid maximal/ extreme twisting or rotation movements post-op. This is partly due to the possible influence of extreme twisting on wound healing.
		Patients ought to avoid maximal/ extreme flexion or bending movements post-op. Most common reasoning identified a need to establish that the fusion was stable and to prevent unnecessary pain.
		Sufficient time must be allowed for bony healing/ fusion to take place before it is safe for an individual to return to certain activities.
		Stages of healing will determine participation in activities.
		The biopsychosocial model is an important framework of care.
		Patient Centred Care is an important framework of care.
		An individual’s anxiety or fear will affect total recovery/ rehabilitation.
		An individual’s beliefs will affect total recovery/ rehabilitation. This might include cultural beliefs, healthcare beliefs, or fear avoidance beliefs.
		Exercise has numerous benefits including promoting recovery, as well as physiological benefits and social benefits.
		Exercise models are an important component of care to both aid physiological function, and sports rehabilitation.
		Pain control and management techniques are important in post-op care and return to activities. This might include analgesia and/ or other modalities such as massage, ice, ultrasound, or hydrotherapy.
		Return to school includes multiple benefits such as academic and/or social integration, normalising routines and behaviours, reducing fear or prevention of being medicalised.
		A graded lifting/ carrying program is an important part of recovery. This program may be time, weight, or position based.
		Information and guidance ought to be provided on benefits and use of sports, exercise, and physical activity following spinal fusion.
		Graded return to sports is important ensuring safety in the post-operative period.
Pre-operative care	Theme of pre-operative care included statements that considered the value and importance of a pre-admission review or education session, a pre-admission home exercise plan, and an understanding of pre-operative or baseline activity levels. Additional comments from Round 3 discussed pre-operative and post-operative handbooks being prepared.	A standardised pre-admission appointment ought to offered for the purpose of setting expectations, timelines of recovery, restrictions, and returning to activities or exercise with the benefit of improving long-term outcomes.
		A standardised pre-admission appointment ought to be offered to help manage fear, psychological preparation and adherence, and what to expect whilst in hospital.
		Pre-operative education is essential in addressing psychological issues & expectations and promoting post-operative rehabilitation
		A pre-admission home exercise plan ought to be offered to optimize function and aid return to sports, exercise, and physical activity with muscular benefits identified.
		It is important to understand the patient’s pre-operative activity levels as these will contribute to total recovery and rehabilitation.
		Seeing physio pre-op helps to promote recovery post-operatively (e.g., prehabilitation or managing expectations).
Physiotherapy treatment modalities	Physiotherapy treatment modalities were primarily exercise based with the highest levels of agreement on patient education being a necessary treatment modality. Additional comments included focus on mobility and core conditioning in early stages with transition to patient specific goals at 2–3 months.	Education.
		Active range of motion or stretching or muscle lengthening work.
		Postural work or core work.
		Strength exercises.
		Walking, mobility, or gait work.
		Cardiovascular fitness.
		Sports specific rehabilitation.
		Return to ADLs and functional goals.
Multidisciplinary team (MDT) members	The MDT was discussed by a number of participants who agreed that family/parents/caregivers, surgeons, physiotherapists, and nursing staff all form an essential part of post-operative care. However, wider members of the MDT and allied healthcare professionals did not reach agreement for routine involvement in post-operative care. Further Round 2 comments explored involvement of the wider MDT when required but not as routine (e.g., Dieticians, Social Workers and Psychologists).	Nursing staff.
		Physiotherapists.
		Surgeons.
		Parents/ family/ caregivers.
		The wider MDT (e.g., Dieticians, Psychologists, Occupational Therapists, Social Workers) are not a standard requirement and only needed if there is a specific clinical need.
Inpatient rehabilitation	Inpatient rehabilitation includes activities completed, and information provided whilst under inpatient care.	Patients ought to be encouraged to commence weightbearing activities on or before Day 1. This includes activities such as standing, getting out into a chair, mobilising and walking.
		Patients ought to be working on their rehabilitation progression whilst in hospital. This inpatient rehabilitation includes activities such as mobility, standing, sitting, stair climbing.
		Patients are given an activity timeline on discharge from inpatient stay to aid return to sports, exercise and physical activities.
		Patients are given a home exercise plan on discharge from inpatient stay to aid return to sports, exercise and physical activities.
Phase 1/ Early content of care	Early phase rehabilitation included items such as water-based exercises, rehabilitation, or hydrotherapy. Wound healing and outpatient appointments being provided in the early stage. Psychological interventions such as goal setting, rapport with treatment provider, as well as motivation were all agreed upon. Additionally, statements around returning to school at 4 weeks post-operatively, surgical clinic reviews, and the role of early phase rehabilitation were agreed upon.	There should be an early phase of rehabilitation that ought to commence somewhere between 0–6 weeks post-operatively.
		Early phase rehabilitation from 0–3 months ought to be a part of post-operative care.
		Light core exercises in the first 6 weeks, increasing activity levels between 6–8 weeks and then further additional activities from 8–12 weeks.
		Early phase activities may focus on exercise, physiotherapy, reducing stiffness and regaining energy levels.
		A post-op [surgeon] clinic review is a necessary component of care. This gives an opportunity to ensure progress, check wound healing, or possibly pain scores.
		A post-op surgical review ought to take place prior to 12 weeks.
		Psychological issues need to be considered/ addressed in post-op care.
		Individuals need to be mentally and/or emotionally ready to commence sports, exercise, or physical activity.
		Patient motivation will affect total recovery and rehabilitation. This may include addressing poor motivation or personal motivation.
		Encouragement/ reassurance regarding the surgery and rehabilitation process is an important part of post-op care. This may be due to the influence of pain or the nature of their pre-operative activity levels.
		Rapport with treatment provider will affect total recovery/ rehabilitation. Good rapport may help with encouragement and progression of exercise.
		Goal setting is an important component of rehabilitation and may help with achievement of milestones and timelines for recovery.
		Wound healing is a milestone both for return to sports and physiotherapy progression.
		A wound check by the healthcare provider is a necessary component of usual care post-op.
		Individuals ought to be encouraged to return to school by approximately 4 weeks post-operatively. This may include a graded return or criteria such as sitting comfortably and off analgesia.
		Hydrotherapy/ water-based rehabilitation/ swimming can only take place once the wound is healed.
		Hydrotherapy/ water-based rehabilitation/ swimming is not essential, but can be offered based on a patients preference and enjoyment of water.
		The first 6 months will establish a graded RTS but there may be variability in time between individuals
		Every patient is unique therefore graded return to sports and rehabilitation protocols will differ between patients
		Restriction of sports/ impact activities in the immediate period post-operatively.
		Ongoing appointments are not routinely necessary but offered on a situational basis, e.g., elite sports or struggling.
		Avoiding maximal ROM for an agreed timeframe post-operatively.
Phase 2/ intermediate	Only 1 statement regarding the intermediate phase of recovery achieved consensus in Round 3. Round 2 and 3 comments highlighted dissonance around definitions of early mid-stage and late-stage phases of care. Further comments discussed having a guide on timeframes rather than a rule and due to variability between patients on what can be achieved within different timeframes.	Mid-stage rehabilitation might include offering a physiotherapy or surgeon appointment.
Phase 3/ late phase	2 statements were agreed upon with regards to late-stage rehabilitation. Firstly, inclusion of a post-operative surgical review. Secondly, rehabilitation focusing on activities of daily living, sport, and work. Dissonance was identified in Round 2 when some participants discussed that non-union is only visible 3–5 years post-operatively, whilst others stated that mechanical hardware issues are often visible between 6 weeks to 6 months post-operatively.	Post-op surgeon review at 12 months is important. This may help with identifying any further concerns, or possibly lead to discharge from surgical care.
		During late-stage rehabilitation exercises are given and might focus on activities of daily living, sport, and work.
Phase 4/ final phase	Only 1 statement achieved consensus with regards to final phase rehabilitation from 12 months post-operatively, stating that there are no restrictions from this point onwards. However, there is an absence of information regarding the possibility or content of physiotherapy or rehabilitation in return to sports, exercise, and physical activity.	There are no restrictions from 12 months onwards.

**Table 6:** Recommendations generated from Round 3 further comments.

Statement	Supporting Comments
Scoliosis specific exercises (SSE) might be appropriate in some cases	“Scoliosis Specific Exercises may be needed but not in everyone”.
	“Re-education on postural and muscular balance and physiological breathing function and activities of daily living (ADL) activities with the guidance of educated possibly physiotherapeutic scoliosis specific exercises certified physiotherapists”.
Post-operative physiotherapy ought to be provided based on need rather than given to every patient	“Similarly, post-op physio needs assessing rather than just something that can be done in everyone as this is not realistic and would waste valuable resource”.
	“Rehabilitation from physiotherapy once discharged home can be done on a patient need basis. Need for input can be identified in clinic on medical review”.
	“Not everyone needs a rehab programme”.
	“Post-surgery we offer specific rehabilitation on patient need basis”.
Timeframes for milestones and rehabilitation will vary between patients and are dependent on a variety of unique factors	“Milestones described ranged from 4 weeks to 12 months. Milestones vary from person to person. It is not possible to set an exact number on the milestones”.
	“I don’t think trying to define mid-stage, intermediate phase, or late-stage rehab is really that helpful. Each patient is going to progress through their rehab based on several unique individual variables and what their personal physical goals are”.
	“This is a vague period that is going to be highly variable between patients”.
Respiratory exercises might be appropriate in some cases as part of rehabilitation	“Respiratory exercises or incentive spirometry is the milestone of healing”.
	“The patients need respiratory exercises”.
Specialist members of the multidisciplinary team can perform post-operative reviews (e.g., physios or nurses)	“Post-operative reviews are important but don’t necessarily need to all be done by the surgeon as a well-trained nurse practitioner, physiotherapist, physician or other member of the team would likely be able to address most patient concerns/questions”.
	“Our service has a 6-week review post op but this is carried out by a specialist nurse or physiotherapist”.

Summary of phases of care within international expert consensus on post-operative rehabilitation for return to sports, exercise and Physical Activity following Spinal Fusion in AIS.

As visualised in Fig 4, there are six themes that follow an iterative flow in line with the patient journey. Each theme has a definition and is detailed in Table 5. Pre-operative care included statements that considered the value and importance of a pre-admission review or education session, a pre-admission home exercise plan, and an understanding of pre-operative or baseline activity levels. Additional comments from Round 3 discussed pre-operative and post-operative handbooks being prepared. Inpatient rehabilitation included activities completed such as sitting in a chair, walking, stair climbing, and information provided whilst under inpatient care. Early phase 1 outpatient rehabilitation included items such as water-based exercises, rehabilitation, or hydrotherapy. Wound healing and outpatient appointments being provided in the early stage. Psychological interventions such as goal setting, rapport with treatment provider, as well as motivation were all agreed upon. Additionally, statements around returning to school at 4 weeks post-operatively, surgical clinic reviews, and the role of early phase rehabilitation were agreed upon. Intermediate Phase 2 rehabilitation had only 1 statement. Round 2 and 3 comments highlighted dissonance around definitions of early mid-stage and late-stage phases of care. Further comments discussed having a guide on timeframes rather than a rule and due to variability between patients on what can be achieved within different timeframes. Late Phase 3 had 2 statements. Firstly, inclusion of a post-operative surgical review and secondly, rehabilitation focusing on activities of daily living, sport, and work. Dissonance was identified in Round 2 when some participants discussed that non-union is only visible 3–5 years post-operatively, whilst others stated that mechanical hardware issues are often visible between 6 weeks to 6 months post-operatively. Only 1 statement achieved consensus with regards to final phase rehabilitation from 12 months post-operatively, stating that there are no restrictions from this point onwards. However, there is an absence of information regarding the possibility or content of physiotherapy or rehabilitation in return to sports, exercise, and physical activity. Throughout the process there were statements on various considerations that are not necessarily time or milestone based. These overarching considerations ought to be considered throughout the rehabilitation process. Considerations that achieved consensus included frameworks such as patient centered or biopsychosocial care, using milestones in rehabilitation, graded lifting, carrying and return to sports, returning to school, promoting exercise, and awareness of an individual’s psychological state. The MDT was discussed by a number of participants who agreed that family, parents, caregivers, surgeons, physiotherapists, and nursing staff all form an essential part of post-operative care. However, wider members of the MDT and allied healthcare professionals did not reach agreement for routine involvement in post-operative care. Physiotherapy treatments were also agreed upon modalities were primarily exercise based with the highest levels of agreement on patient education being a necessary treatment modality. Additional comments included focus on mobility and core conditioning in early stages with transition to patient specific goals at 2–3 months.

During Round 3 there was a final opportunity for open comments. The qualitative data captured some further issues that are still important for participants at the end of the Delphi process. A further 5 recommendations were generated from these open comments with agreement >1 participant (Table 6). At the end of the consensus processes ongoing issues discussed in comments included scoliosis specific exercises, post-operative rehabilitation, timeframes, respiratory exercises, and involvement of specialist members of the MDT in post-operative reviews.

## Discussion

This study was the first to establish a consensus on timelines, milestones, and content of rehabilitation for return to sports, exercise, and physical activity following spinal fusion in AIS. This Delphi generated a series of consensus statements that relate to nine major themes as well as the role of the MDT and philosophical approaches to care. However, further subgroup analysis demonstrated consensus among surgeons and divergence among physiotherapists highlighting the need for further exploration of these statements.

### Expert consensus on timelines and milestones

A large number of statements were generated supporting rehabilitation in the initial and early phases of care. However, the Intermediate Phase 2, Late Phase 3, and Final Phase 4 stages of rehabilitation in return to sports, exercise, and physical activity remain underrepresented with 1 statement for both Intermediate and Final and 2 statements for Late phase rehabilitation. This demonstrates dissonance in relation to earlier statements regarding the need for a graded return to sports and sports specific rehabilitation. The 2016 consensus statement on return to sport describes a continuum paralleled with recovery and rehabilitation with three phases (1) return to participation, (2) return to sport, and (3) return to performance [80]. However, beyond Early Phase 1 rehabilitation this Delphi consensus lacks further detail with regards to return to sport and performance, thereby warranting future exploration in late-stage sports, exercise, and physical activity post-operatively. Graded return to sports is a complex multifactorial process and has been recognised as a detailed part of rehabilitation [81]. There is limited evidence surrounding physiotherapists or other medical or healthcare professionals’ confidence or knowledge regarding administration of a return to sport program, particularly within spinal pain. However, one study concluded that a lack of knowledge and training was a barrier to implementing psychological strategies in return to sports rehabilitation [82]. Further, exploration of physiotherapists’ confidence, knowledge, content, and application of return to sports programs is necessary, particularly following spinal fusion in AIS. Furthermore, this Delphi highlighted vagueness with regards to timeframes for return to sports, exercise, and physical activity. This is likely to make it more acceptable to professionals involved in the rehabilitation and return to sports, exercise, and physical activities in allowing individual tailoring of guidance in accordance with the specific and complex biopsychosocial needs of the patient [83].

### MDT involvement

The role of the MDT was a key objective of this study as SOSORT recommendations support the involvement of the wider MDT [12]. However, which members of the MDT ought to routinely be involved in care, particularly when supporting post-operative return to sports, exercise, and physical activity was not defined [21,22]. Experts highlighted a wide variety of MDT members who may not be routinely included in post-operative rehabilitation but have a valuable role in supporting care if there is a specific clinical need. This is the first study to explore who from the MDT ought to be involved in post-operative return to sports, exercise, and physical activity in AIS. Other literature has evaluated the benefits of the MDT in contexts such as cancer care and primary care with evidence demonstrating an improved quality of care [84–86]. However, further work is needed exploring the involvement and benefits of the MDT in AIS. Parents, family, or caregivers were also determined as having a necessary involvement in post-operative rehabilitation. Although not a part of the professional MDT literature shows that parents experience substantial emotional burden regarding their child’s future, work opportunities, ongoing back problems and significant concern around consenting for spinal fusion surgery [87,88]. Furthermore, parents may be more worried than their child following a diagnosis of AIS and literature shows that a parent’s response can either promote or confound a child’s health outcomes [87,89]. Therefore, this consensus supporting the involvement of parents, family and caregivers is in keeping with the literature demonstrating the value and benefit of parental information and support on a child’s post-operative outcomes [88,89].

### Philosophical approaches to rehabilitation

This study set out to explore philosophical approaches underpinning post-operative rehabilitation. Both the biopsychosocial model and patient centered care were agreed upon as necessary frameworks providing a philosophy of care that guide the application of medical knowledge based on the needs and of and shared decision making with the patient [90,91]. Within this Delphi consensus are a series of statements on overarching considerations that ought to be considered at each stage of rehabilitation and can be selected based on patient needs and choice. Additionally, there are several statements that refer to the possible use of an intervention at a specific stage based on the patients needs. Although, the biopsychosocial model has been criticized for its vagueness, it allows clinicians to broaden their gaze to a variety of factors that may influence return to sports, exercise and physical activity [90]. Together both philosophical approaches allow clinicians to select and tailor appropriate interventions based on patient need and choice rather than a strict prescriptive guideline, improving application and outcomes of rehabilitation [91,92].

### Strength of agreement in consensus

Study findings demonstrated moderate agreement (W = 0.5, p < 0.05) [67]. Although, we set out to achieve strong agreement (W = 0.7) Kendall’s coefficient is designed to denote the strength of the agreement rather than being used as an exact cut off point [67]. Furthermore, the presence of moderate agreement (W = 0.5) alongside the significance of the p value demonstrates concordance between experts [67,93]. The definition of consensus is contested in the literature and has not yet been established [55]. However, level of agreement between experts is more easily defined with many studies using percentage agreement, particularly when Likert scales have been used [55]. Our study first evaluated percentage agreement with consensus >75% (Strongly Agree or Agree), followed by Kendall’s coefficient of concordance demonstrating both strength of agreement and significance [65]. It was decided in discussion with the study steering group that the presence of moderate agreement (W = 0.5) and significance in results (p < 0.05) provided an appropriate stopping criterion and a fourth round was not required [55]. Furthermore, the challenges to a fourth round include participant attrition resulting in overestimation of consensus, particularly if participants with minority perspectives drop out [94,95]. Further research may usefully explore agreement distribution and has the potential to offer new perspectives, explore the multifactorial nature of post-operative rehabilitation, or offer a deeper understanding of factors influencing agreement amongst experts [96].

### Stability of consensus statements

For all statements stability was calculated between round 2 and round 3. The Wilcoxon sum rank test demonstrated no significant changes and experts were consistent in their views (p > 0.05) [97]. Furthermore, when performing multiple tests it is important to consider the possibility of Type 1 error and therefore need for a Bonferroni correction [97]. In our study all statements were not significant therefore the addition of a Bonferroni correction did not change the results of the Wilcoxon sum rank test for the sixty-three statements that were rated on the Likert in both Round 2 and 3 (p = 0.0008), for the fifty-three statements that were both rated on the Round 2 and 3 Likert and had > 75% agreement (p = 0.001). Therefore, the Bonferroni correction was not necessary as all values were p > 0.05 (not statistically significant).

### Recommendations on the role of scoliosis specific exercises

Scoliosis Specific exercises (SSE) including Schroth are tailored exercises focusing on the three-dimensional treatment of AIS but realigning the spine, rib cage, shoulders, and pelvis [98]. There are a variety of different schools that teach exercises that come under the definition of SSE and are recommended by SOSORT [12,98]. The effectiveness of SSE remains debated with one review reporting a short-term improvement in Cobb angle, trunk rotation, and quality of life [99], although the improvement in Cobb angle did not exceed the minimum clinically important difference [99]. Meanwhile, another review concluded the evidence is insufficient to confirm the benefits of one specific physiotherapy intervention over another [100]. The opportunity for further comments in this Delphi revealed that there is still debate amongst experts regarding SSE, with dissonance between participants convicted in their views. A statement around the use of SSE was generated from Round 1 and participants had the opportunity to vote on this during Round 2 (data in S1 Appendix). During Round 2 and 3 Likert voting SSE was dropped due to a lack of consensus but re-introduced with open comments >1 participant (Table 6). This dissonance is perhaps reflective of the wider debate in the literature regarding the role of SSE [100]. Furthermore, in post-operative spinal fusion, the Cobb angle has been objectively reduced through mechanical instrumentation [101]. Therefore, unless there is presence of scoliotic curves outside the area of instrumentation, the requirement of SSE to reduce the size of a spinal deformity is no longer present [102]. Consequently, the justification for the use of SSE post-operatively is reduced to only operated patients with pain to increase function as stated in SOSORT guideline [12,103]. However, further exploration of the statement generated by open comments is required to understand rationale for comments and application [96].

### Recommendations on role of respiratory exercises

Spinal fusion in the treatment of scoliosis is known to have varying effects on respiratory function due to the effect on the chest wall, particularly with an anterior approach or thoracotomy [104]. Posterior spinal fusion is thought to be less deleterious on post-operative respiratory function, with debate in the literature whether posterior fusion increases or maintains respiratory function [104–106]. Statements around respiratory care became another example of dissonance with statements being regarding the role of respiratory exercises being included during round 2 alongside contrary statements stating that they should not be routinely offered, then both statements being discarded during round 3 – highlighting dissonance. During round 3 one final statement (‘respiratory exercises might be appropriate in some cases as part of rehabilitation’) was generated from open comments. Although, this final statement is more vague with regards to respiratory care, it may be appropriate in the context of other literature which suggests that the surgical approach chosen in addition to the stage of adolescence and growth mean that multiple factors will influence respiratory function [104,107]. Therefore, this dissonance warrants the need for further exploration of respiratory exercises as part of post-operative rehabilitation in AIS. Furthermore, aerobic and resistance training post-operatively have known benefits on respiratory function when compared to aerobic training alone [108]. Clarity is needed on where these consensus statements regarding respiratory exercises fit with an aim of facilitating sports, exercise, and physical activity.

### Experts included within the study

Included within the surgical experts were three experts registered as physiatrists, medically qualified practitioners holding a medical license to practice. However, only one participant stated this at the start of the Delphi process. This study contained one open question asking participants to describe their practice, but did not explore the specific activities undertaken by experts and Delphi definitions of experts remains contentious and poorly defined [49]. However, the literature discusses experts having the experience and knowledge of the issue and willingness and capacity to participate [49]. Therefore, in discussion with the study steering group, it was determined that physiatrists, being medically qualified, met the definition of an expert, would be included in subsequent rounds and calculation of consensus. These individuals were counted within our surgical experts but it was unclear from the data whether these participants had dual accreditation as both medical doctors and physiotherapists or the extent to which their practice included a surgical component. However, it is widely accepted that many surgeons divide their time between activities such as clinics, teaching, and research [109]. The proportion of surgeons who undertake operations and the portion of time spent doing so remains unknown [109]. physiatrist responses were often more closely aligned with physiotherapist responses rather than surgeon responses, perhaps due to their training involving specific rehabilitation expertise [110]. However, although physiatrist numbers are increasing, due to small numbers in this study it was not possible to demonstrate these differences with statistical testing [111].

### Strengths and limitations

This international Delphi had several strengths, being the first study to establish expert surgeon and physiotherapy consensus on content of, philosophical approach, and MDT involvement in return to sports, exercise and physical activity following spinal fusion. Throughout this study all methodological considerations were discussed and agreed upon with patient and public involvement (PPI) representative (ER), and the study steering group (AS, NH, AG, AR). This study has been reported in accordance with Guidance on Conducting and REporting Delphi Studies guidelines as recommended by the Enhancing the QUAlity and Transparency Of Health Research network. All methods were described as per study protocol and subject to peer review process [33]. Expert consensus has been established using inductive content analysis for the presence of themes, patterns or concepts followed by statistical testing to determine agreement and consensus in the group.

However, there were also some limitations to this Delphi study. All consenting eligible experts were able to participate regardless of whether they work part time or only work with conservatively managed individuals, this included 3 physiatrists within the surgical group although physiatrists as a unique profession was not individually sought. This study did not set out to involve other members of the MDT who may have valuable insight into post-operative rehabilitation. Due to small numbers of experts from the continents of Africa and South America and lacking information about hospital system, culture, and participant distribution it was not possible to evaluate dissonance globally. Finally, this study did not specify variables such as anterior or posterior surgical approach, or stage of growth when undergoing fusion which may influence components of rehabilitation, and return to sports, exercise, and physical activity.

### Implications for practice and research

This Delphi has successfully established an expert consensus from pre-operative care until 12 months post-operatively where there are no limitations on sports, exercise and physical activity participation. However, there remains a lack of detail, particularly regarding rehabilitation from 3 months post-operative onwards. Therefore, further development of consensus regarding the later phases of rehabilitation beyond 3 months post-operatively is required in the form of future pilot or feasibility testing of novel physiotherapeutic rehabilitation interventions for post-operative rehabilitation in AIS [36,43,51,52]. As discussed this Delphi highlighted areas of dissonance between experts, namely SSE and respiratory exercises, therefore future exploration of dissonance with experts may offer additional insights or reasons for diverging opinions such as demographic, cultural, or professional differences between individuals [34]. A Delphi consensus does not replace original research and further development is required to form clinical guidelines regarding return to sports, exercise, and physical activity [52].

## Conclusion

This study was the first to generate an international consensus on return to sports, exercise, and physical activity following spinal fusion in AIS. A total of 71 consensus statements were generated that followed a series of themes that start with pre-operative care and end with final phase 4 rehabilitation and no restrictions at 12 months post-op. The latter stages of rehabilitation from intermediate to final phase lack detail with regards to returning to sports, exercise, and rehabilitation and warrant further development.

## Supporting information

S1 Appendix Round 1 themes and statements distributed in Round 2. (DOCX)

S2 Appendix Round 2 statements from Likert rating and open comments >1 participant. (DOCX)

S1 File GRIPP2 Short Form. (DOCX)
