# Supplementary material for: International consensus on sports, exercise, and physical activity participation during post-operative interventions for Adolescent Idiopathic Scoliosis: An e-Delphi study
Source: PLoS One. 2026 Feb 23;21(2):e0322346. doi: 10.1371/journal.pone.0322346 (PMC12928444; doi:10.1371/journal.pone.0322346)
Supplement: S2 Appendix — (DOCX) [file pone.0322346.s003.docx]

**S3 Supplementary File 1.** GRIPP2 Short Form

| **Section and topic** | **Item** | **Reported on page No** |
| --- | --- | --- |
| 1: Aim | The aim of PPI was to enhance the quality and appropriateness of the study . PPI representative (ER) and the study steering group (AS, NH, AR, AG) contributed, reflected, and discussed throughout the research process to improve the relevance and quality of the study . | 8-9,  13-14 |
| 2: Methods | PPI has been involved in this study from conceptualisation until dissemination. PPI representative (ER) who was part of the study steering group along with academics and clinicians (AS, NH, AR, AG) has given feedback on the study protocol, questionnaire development, participant information sheets, consent forms, results analysis, and the final manuscript. | 8-9,  13-14 |
| 3: Study results | The PPI representative (ER) and study steering group (AS, NH, AR, AG) have determined that this research is both useful and justified from a patient population perspective The steering group for this study and PPI representative have discussed all outcomes during results analysis and presentation. | 8-9,  13-14 |
| 4: Discussion and conclusions | Both PPI and study steering group feedback has been considered throughout the process with influence from the study steering group with regards to statements that caused dissonance amongst experts. | 8-9,  13-14 |
| 5: Reflections / critical perspective | PPI representative (ER) supports and agrees with the importance of and necessity of this work. | 13-14  37 |
