# Supplementary material for: International consensus on sports, exercise, and physical activity participation during post-operative interventions for Adolescent Idiopathic Scoliosis: An e-Delphi study
Source: PLoS One. 2026 Feb 23;21(2):e0322346. doi: 10.1371/journal.pone.0322346 (PMC12928444; doi:10.1371/journal.pone.0322346)
Supplement: S1 File — (DOCX) [file pone.0322346.s001.docx]

**S1 Appendix 1.** Round 1 themes and statements distributed in Round 2

| **Theme** | **Statements** |
| --- | --- |
| Theme 1:Graded Return to Sports | 1) A Graded RTS is important and beneficial in progressing up to contact sports, promoting recovery and function. This could be milestone based or time based. |
|  | 2) There are no restrictions post-op affecting RTS. Participants should return to all activities as soon as possible or as soon as they feel able. |
|  | 3) Patients are always restricted post-op from returning to certain activities such as impact or extreme sports. |
|  | 4) Early phase rehabilitation from 0-3 months ought to be a part of post-operative care. This might include light core exercises in the first 6 weeks, increasing activity levels between 6-8 weeks and then further additional activities from 8-12 weeks. |
|  | 5) Intermediate phase rehabilitation from 4-6 months ought to be part of post-operative care. |
|  | 6) Late phase rehabilitation from 6-12 months ought to be a component of rehabilitation which may or may not include contact sports. |
|  | 7) Final Phase from 12months + is a milestone of post-operative rehabilitation. There are no restrictions from this point onwards. |
| Theme 2: Physiotherapy Timeframes | 8) Time based milestones are an important consideration in physiotherapy progression. Milestones described ranged from 4 weeks to 12 months. |
|  | 9) There should be an early phase of rehabilitation that ought to commence somewhere between 0-6 weeks post-operatively. Activities may focus on early exercises, physiotherapy, reducing stiffness and regaining energy levels. |
|  | 10) A mid-stage rehabilitation component ought to be offered between 6-12 weeks. This might include offering a physiotherapy or surgeon appointment. |
|  | 11) A late-stage rehabilitation component ought to be offered from 12 weeks onwards where exercises are given and might focus on ADLs, sport, and work. |
|  | 12) Ongoing rehabilitation or appointments ought to be considered long term. This might include core strengthening, or regular time-based appointments depending on need. |
| Theme 3: Physiotherapy Interventions / Modalities | 13) No routine physiotherapy should be offered |
|  | 14) Education |
|  | 15) Active ROM or stretching or muscle length work |
|  | 16) Postural Work or Core Work |
|  | 17) Strength exercises |
|  | 18) Walking, Mobility, or Gait Work |
|  | 19) Cardiovascular fitness |
|  | 20) Manual Therapy / hands on treatment |
|  | 21) Schroth or Scoliosis Specific Exercise Programs |
|  | 22) Balance or Proprioceptive work |
|  | 23) Sports specific rehab |
|  | 24) Pilates |
|  | 25) Adjuncts to physiotherapy ought to be offered |
|  | 26) Return to ADLS and functional goals |
| Theme 4: Movement Safety | 27) Patients ought to avoid maximal / extreme twisting or rotation movements post-op. This is partly due to the possible influence of extreme twisting on wound healing. |
|  | 28) Patients ought to avoid maximal / extreme flexion or bending movements post-op. Most common reasoning identified a need to establish that the fusion was stable and to prevent unnecessary pain. |
|  | 29) Criteria for returning to maximal range of motion is time based. Timeframes may range from 2-3 weeks until 6 months. |
| Theme 5: Bony Fusion | 30) Sufficient time must be allowed for bony healing / fusion to take place before it is safe for an individual can return to certain activities. |
| Theme 6: Multidisciplinary Team Involvement | 31) A full MDT approach is an important aspect of post-op care following spinal fusion in AIS |
|  | 32) Nursing staff |
|  | 33) Pain Team |
|  | 34) Physiotherapists |
|  | 35) Surgeons |
|  | 36) Parents / family / caregivers |
|  | 37) Dieticians |
|  | 38) Occupational therapists |
|  | 39) Social worker |
|  | 40) Psychologists |
|  | 41) Orthotist |
|  | 42) Wider medical team of doctors E.g. Primary Care and medical team |
|  | 43) Support groups |
| Theme 7: Surgeon Involvement | 44) A post-op clinic review is a necessary component of care. This gives an opportunity to ensure progress, check wound healing, or possibly pain scores. |
|  | 45) The surgeon ought to advise when an individual is safe to return to sports, exercise, and physical activities. |
|  | 46) Post-op surgeon review at 6 weeks is important. This may include assessing progress, further guidance or cautions as necessary. |
|  | 47) An X-ray at 6 weeks post-op is an important to monitor correction and in some cases will be done prior to RTS. |
|  | 48) Post-op surgeon review at 12 months is important. This may help with identifying any further concerns, or possibly lead to discharge from surgical care. |
|  | 49) Surgeon review at 2 years is important and might help with identifying any further concerns. |
| Theme 8: Frameworks | 50) The biopsychosocial model is an important framework of care |
|  | 51) Patient Centred Care is an important framework of care |
|  | 52) No formal models, frameworks, or approaches are needed in the post-op management of AIS |
| Theme 9: Psychological issues | 53) Psychological issues need to be considered / addressed in post-op care. |
|  | 54) An individual’s anxiety or fear will affect total recovery / rehabilitation. |
|  | 55) Individuals need to be mentally and/or emotionally ready to commence sports, exercise, or physical activity. |
|  | 56) Patient motivation will affect total recovery and rehabilitation. This may include addressing poor motivation or personal motivation. |
|  | 57) An individual’s beliefs will affect total recovery / rehabilitation. This might include cultural beliefs, healthcare beliefs, or fear avoidance beliefs. |
|  | 58) Encouragement / reassurance regarding the surgery and rehabilitation process is an important part of post-op care. This may be due to the influence of pain or the nature of their pre-operative activity levels. |
|  | 59) A cognitive behavioural approach ought to be taken in post-op care in AIS. This may aid behaviour change and postural correction. |
|  | 60) Rapport with treatment provider will affect total recovery / rehabilitation. Examples include good rapport may help with encouragement and progression of exercise. |
|  | 61) Goal setting is an important component of rehabilitation and may help with achievement of milestones and timelines for recovery. |
| Theme 10: Pre-operative Care | 62) A standardised pre-admission appointment is important for the purpose of setting expectations for specific reasons including; timelines for recovery, managing fear, psychological preparation and adherence, what to expect post-operatively whilst in hospital, restrictions, and returning to activities or exercise with the benefit of improving long-term outcomes. |
|  | 63) A pre-admission home exercise plan ought to be offered to optimize function and aid return to sports, exercise, and physical activity with muscular benefits identified. |
|  | 64) It is important to understand the patient’s pre-operative activity levels as these will contribute to total recovery and rehabilitation. |
| Theme 11: Exercise benefits | 65) Exercise has numerous benefits including promoting recovery, as well as physiological benefits and social benefits. |
|  | 66) Exercise Models are an important component of care to both aid physiological function, and sports rehabilitation. |
| Theme 12: Inpatient Rehabilitation | 67) Usual care consists of approximately 5 days in hospital with the discharge criteria including pain control and bowels opened. |
|  | 68) Patients ought to be encouraged to commence weightbearing activities on or before Day 1. This includes activities such as standing, getting out into a chair, mobilising and walking. |
|  | 69) Patients ought to be working on their rehabilitation progression whilst in hospital. This inpatient rehabilitation includes activities such as mobility, standing, sitting, stair climbing. |
|  | 70) Patients are given an activity timeline and/or home exercise plan on discharge from inpatient stay to aid return to sports, exercise and physical activities. |
| Theme 13: Wound Healing | 71) Wound healing is a milestone both for return to sports and physiotherapy progression. |
|  | 72) A wound check by the healthcare provider is a necessary component of usual care post-op. |
| Theme 14: Nurse Review | 73) Nursing review ought to be scheduled at regular time intervals post-op. This might include a telephone review and/or face to face appointments. |
| Theme 15: Pain | 74) Pain control and management techniques are important in post-op care and return to activities. This might include analgesia and / or other modalities such as massage, ice, ultrasound, or hydrotherapy. |
|  | 75) Pre-op level of pain has an effect on an individual’s ability to participate in rehabilitation post-op. |
|  | 76) Reduced pain is a necessary milestone before an individual can return to sports, exercise, and physical activity. |
|  | 77) Reduced or controlled pain is a necessary milestone before progression of physiotherapy modalities or exercises. |
|  | 78) Pain needs to be adequately managed or reduced prior to physiotherapy discharge. |
| Theme 16: Return to School | 79) Individuals ought to be encouraged to return to school by approximately 4 weeks post-operatively. This may include a graded return or criteria such as sitting comfortably and off analgesia. |
|  | 80) Return to school includes multiple benefits such as academic and/or social integration, normalising routines and behaviours, reducing fear or prevention of being medicalised. |
| Theme 17: Carrying Capacity | 81) A graded lifting / carrying program is an important part of recovery. This program may be time, weight, or position based. |
| Theme 18: Swimming, Hydrotherapy, and Water based rehabilitation | 82) Participation in swimming, hydrotherapy, or water-based exercise / rehabilitation is an important treatment modality post-op facilitating return to sports, exercise, or physical activity. |
|  | 83) Individuals ought to commence water-based exercise / rehabilitation at 6 weeks post-op |
|  | 84) Individuals can commence water-based exercise / rehabilitation or hydrotherapy as soon as the wounds are healed (approx. 2 weeks post-op). |
| Theme 19: Respiratory Care | 85) Respiratory / breathing exercises are a necessary component of rehabilitation. This might include respiratory or breathing exercises, incentive spirometry, chest expansion. |
